# Tributyltin chloride alters the structural, genomic, and epigenomic integrity of postejaculatory mammalian sperm

**DOI:** 10.1080/15592294.2025.2552129

**Published:** 2025-09-15

**Authors:** Muhammad S. Siddique, Santosh Anand, Marie-Pierre L. Gauthier, Jason O. Brant, Michael P. Kladde, Ramji K. Bhandari, Bradford W. Daigneault

**Affiliations:** a Department of Animal Sciences, University of Florida, Gainesville, FL, USA; b Department of Biological Sciences, University of Missouri, Columbia, MO, USA; c Department of Biochemistry and Molecular Biology, College of Medicine, University of Florida, Gainesville, FL, USA; d Department of Biostatistics, College of Public Health & Health Professions, University of Florida, Gainesville, FL, USA; e Department of Large Animal and Clinical Sciences, College of Veterinary Medicine, Gainesville, FL, USA

**Keywords:** Bovine, embryo, tributyltin chloride, methylome, sperm

## Abstract

A global priority for ameliorating male factor infertility includes identification of environmental factors and mechanisms that impact sperm function. Detection of endocrine disrupting chemicals (EDC) in seminal plasma and within the female reproductive tract has created an urgent need to understand how environmental stressors alter postejaculatory sperm function. Tributyltin chloride (TBT) is an EDC and epigenetic modifier that causes reproductive disorders. The consequences of TBT exposure on postejaculatory sperm remain unknown. The present study was aimed at identifying structural, genomic, and epigenomic consequences of TBT exposure to postejaculatory sperm. Bovine sperm were exposed to TBT (0, 1, 10, 100 nM) for 24 h followed by quantification of sperm kinematics, DNA integrity, and methylation status. No differences were detected in sperm kinematics or capacitation status. However, acrosome integrity was compromised at both 0 and 24 h (*P* ≤ 0.05). Sperm DNA integrity was also negatively affected after 24 h. Whole-genome methyl-seq revealed ~750 differentially methylated regions (DMRs) associated with exposure to TBT. Ingenuity Pathway Analyses and Gene Ontology identified embryo development, cell signaling, and transcriptional regulation as the most relevant bio-functions of TBT altered DMRs. In conclusion, postejaculatory mammalian sperm exposure to TBT negatively affected parameters important for sperm function while altering DNA integrity and the methylation profile of gene promoter regions. Consequences of sperm exposure to TBT included cellular and molecular mechanisms that are important for sperm function but remain undetected by routine clinical analyses. These findings provide new insight into environmental impacts on postejaculatory sperm structure and function.

## Introduction

Male factor infertility is a global concern affecting approximately 50 million couples worldwide while accounting for nearly half of all idiopathic subfertility in humans [1,2]. Endocrine disrupting chemicals (EDCs) are globally prevalent and their presence in the environment is associated with reproductive disorders including male infertility [3]. Despite international bans on EDCs like tributyltin chloride (TBT), this organotin (OT) remains environmentally stable and a concern for human health including reproductive processes [4]. TBT is one of the most common and widely used anthropogenic OTs present in fungicides and biocides with detection in seafood and fruits of Asian origin shipped to the United States [5]. TBT is well-described for negative influences on male and female reproductive physiology including obesogenic, developmental, and reproductive irregularities in female mice such as reduced ovarian reserve and decreased number of uterine glands [6,7]. TBT also causes male infertility with congenital defects such as cryptorchidism that impair spermatogenesis and negatively regulate seminal parameters including volume, motility, viability, and morphology [8–10]. Despite an abundance of data describing the negative effects of TBT on sperm at the testicular level, there is a significant knowledge gap regarding the influences of TBT on postejaculatory sperm function following acute exposure. Our group previously reported that short-term exposure of bovine sperm to TBT for 90 min reduced motility, mitochondrial function, and subsequent embryo development [11]. Although studies on TBT exposure to postejaculatory sperm appear to be extremely limited, others have reported similar *in vitro* studies with tetrahydrocannabinol (THC) exposure to bovine sperm that impacted fertilization, capacitation, pre-implantation *in vitro* embryo development, and miRNA abundance [12,13]. Similarly, exposure of bovine sperm to Bisphenol (BPA) increased sperm hyperactivity, decreased acrosome reaction levels, and decreased blastocyst rates including cell counts and DNA fragmentation [14].

Chromatin of mammalian spermatozoa is largely protected from environmental insult due to highly condensed DNA packaging during spermatogenesis, yielding heterochromatin structures as a result of histone replacement by protamines. Mature sperm are transcriptionally inactive and translationally repressed [15–17]. The compact chromatin is seemingly required for protection of genetic material during sperm transport through both the male and female reproductive tract [17]. However, approximately 8% of the human and bovine sperm chromatin structure remain open as euchromatin [18], resulting in regions that are presumably accessible and, therefore, susceptible to environmental insult during sperm transport. Sources of postejaculatory sperm exposure to environmental stressors such as EDCs and OTs include seminal plasma [8,9,19] and fluids of the female reproductive tract [6,7,20]. Multiple EDC and OT targets localized on postejaculatory sperm are of significant concern for sperm function and fertility due to independent and congruent functional roles of membrane, acrosomal, mitochondrial, and nuclear integrity that are required for unassisted fertilization. Likewise, the sperm epigenome contains important information that although likely inconsequential for sperm function prior to fertilization, plays critical regulatory zygotic roles that influence early embryo development [21]. Established mechanisms of OT interaction with sperm DNA and TBT-induced epigenetic mechanisms in somatic cells further emphasize the need to explore consequences of TBT exposure with postejaculatory sperm function [9,22,23]. EDCs can affect germ cells by eliciting changes to DNA methylation during critical developmental timepoints of gametogenesis resulting in transgenerational phenotypes by eliciting newly imprinted sites that are transmitted to the next generation through sperm [24,25]. Environmental epigenetic modification refers to explicit change in phenotype triggered by environmental stimuli through epigenetic mechanisms. Environmental factors such as EDCs can influence the epigenome of sperm, resulting in phenotypic variation that is involved in many biological processes including epigenetic transgenerational inheritance carried through germline modification [26,27].

EDCs can also act as novel carboxylates that interact with sperm DNA resulting in a heritable phenotype [28]. For example, testicular sperm exposure to bisphenol A results in changes to DNA methylation, alteration in transcript abundance of epigenetic remodeling enzymes, and variation in histone modification. These epigenomic landscape changes in sperm result in altered transcription of genes that have a profound effect on neural system development, cancer cell proliferation, imprinted regions, and subsequently affect transgenerational inheritance [29,30]. Aberrant DNA methylation of sperm is associated with imprinting disorders and subfertility. Mechanisms underpinning these phenotypes have been attributed to aberrant transcription and DMRs necessary for fertility that are involved in spermatogenesis and early embryonic development [31,32].

Environmentally persistent EDCs can have a profound effect on testicular sperm, but consequences of acute exposure on postejaculatory sperm have not been fully explored. Therefore, the present study aimed to determine mechanistic, structural, and epigenomic consequences of TBT exposure to postejaculatory sperm under the hypothesis that TBT induces subtle phenotypic changes associated with male factor infertility using bovine as a translational model for humans.

## Methods

Experiments conducted herein utilized commercially available frozen-thawed bull sperm from ORIgen (Huntley, MT, USA). Live animals were not used for these experiments. Unless otherwise stated, all reagents were purchased from Sigma Aldrich® (St. Louis, MO, USA).

### Isolation of bovine sperm

Frozen-thawed sperm from two different commercial bulls were used for experiments, while a single bull was used for whole-genome enzymatic methyl-sequencing. In each replicate, four frozen straws were thawed at 37°C for 30s followed by isolation on a discontinuous Percoll gradient (90:45%) and washed twice by centrifugation in HEPES buffered saline in accordance with established protocols [33]. Sperm pellets were then resuspended to a final concentration of 20 × 10^6^ total sperm/mL in 400 µL volumes of a non-capacitating Tyrode’s albumin lactate pyruvate (TALP) medium free of bicarbonate and albumin (99 mM NaCl, 24.8 mM NaHCO_3_,10 mM HEPES, 0.33 mM NaH_2_PO_4_, 24 mM) sodium lactate (60%), 2.4 mM MgCl_2_ ∙ 2 H_2_O, 2.6 mM CaCl_2_2 H_2_O [11].

### Preparation of TBT for sperm exposure

A stock solution of tributyltin chloride 50 mM (Sigma 442,869) was prepared with 100% dimethyl sulfoxide (DMSO). From that 50 mM solution, an additional 1 mM stock solution was prepared using 100% DMSO. Working solutions included three serial dilutions (100, 10, and 1 µM) in 100% DMSO from the 1 mM stock solution. Serial dilutions were stored in glass scintillation vials and wrapped in paraffin to avoid evaporation for storage at room temperature. On the day of experiment, 10 µL of each serial dilution was added to 90 µL of TALP medium to obtain 10,000 nM, 1000 nM, and 100 nM working dilutions of final TBT concentration in 10% DMSO. Then, 1:100 final dilutions in TALP were made to achieve final TBT concentrations of 100 nM, 10 nM, and 1 nM in 0.1% DMSO with media containing sperm. A vehicle control (VC – DMSO 0.1% only) was also included and prepared in the same manner but without TBT. The range of TBT concentrations was based on previously described doses determined as environmentally or physiologically relevant and non-toxic to sperm [11, 34–38].

### Sperm exposure to TBT

Sperm samples were maintained at a final concentration of 20 × 10^6^ total sperm/mL in TALP and were exposed to three concentrations of TBT (100 nM, 10 nM, and 1 nM). All samples were maintained for up to 24 h at ambient (25°C) temperature in non-capacitating conditions to replicate temporal aspects of sperm transport and exposure time within the female reproductive tract and to more accurately replicate sperm interactions with seminal fluid, thereby increasing biological relevance. An aliquot of each sample was warmed to 37°C for 10 min prior to kinematic analyses.

### Sperm kinematic assessment

Sperm kinematic parameters were objectively assessed by computer assisted sperm analysis (CASA-IVOS System, Hamilton Thorne, Beverly, MA). Time points consisted of 0 h (within 5–10 min of TBT exposure), and again at 24 h following maintenance at ambient temperature as previously described. Parameters acquired include total motility (TM), progressive motility (PM), average path velocity (VAP), and curvilinear velocity (VCL). A minimum of five fields and a total of 500 spermatozoa were counted on a heated stage at 37°C by acquiring 30 frames at a rate of 60 Hz with a minimum contrast of 15 and minimum cell size of 10 pixels. Progressive minimum cutoff values included VAP at 20 µm/S, slow cell cut off VAP at 5 µm/S, and VCL cut off of 6 µm/S. All CASA measurements were acquired using Leja 4 chamber 20 µM slides with the addition of 3 µL of sample in each chamber. (IMV 0,251,070). For each experiment, four replicates per bull were performed.

### DNA integrity measurement by terminal deoxynucleotidyl transferase dUTP nick end labeling (TUNEL ASSAY)

TUNEL was used to quantify DNA integrity following sperm exposure to TBT. A One-step TUNEL in situ Apoptosis Kit (Green-FITC) (Elabscience, Cat No. E-CK-A320) was used for this purpose. Sensitivity and specificity of the kit were determined with the use of controls consisting of no enzyme (negative) and the addition of DNase I (positive) to ensure ≥85% labelling in exposed sperm. In this assay, 50 µL of sperm samples previously maintained at a concentration of 20 × 10^6^ total sperm/mL were placed on a glass slide at 37°C for air drying. Paraformaldehyde (4%) was then added to each sample in 100 µL volumes for fixation at a room temperature for 30 min followed by PBS washing. Sperm were then permeabilized by incubation with TritonX 0.1% for 15 min at 37°C followed by washing with phosphate buffered saline, pH 7.3 (PBS). After washing, 100 µL of TdT equilibration buffer was added to each slide and incubated for 30 min at 37°C followed by the addition of 50 µL of labeling solution (TdT equilibration buffer, labeling solution, and TdT enzymes) and incubation again at 37°C for 4 h in a dark, humidified chamber. Slides were then washed with PBS and 100 µL of DAPI working solution (25 µg/mL) was added for 15 min at room temperature followed by a PBS wash. Slides were air dried and analyzed immediately or held at 4°C for future analysis. Four replicates for each bull (2 bulls) were performed with a minimum of 120 sperm per slide enumerated per replicate for a total of 960 sperm for each treatment. Sperm were classified as Not -Fragmented DNA (lack of green FITC fluorescence with DAPI counterstain) Fragmented DNA (green fluorescence encompassing entire head), and Partial fragmentation (both FITC and DNA fluorescence in the merged field) [39–41]. Fluorescence emission and detection were performed at wavelengths of 360/432 nm (DAPI), and 461/515 nm (FITC), respectively. Emission was detected with an AURAIII-UCGRnIR Light Engine (LED Lumencor 4-channel) compatible with Penta band filter cube on a Nikon TE2000U inverted microscope at 200x magnification using Nikon Elements software. We

### Detection of acrosome integrity and capacitation status

Acrosome integrity and capacitation status of sperm were quantified following sperm isolation and TBT exposure as described above. All washes were performed three times at room temperature with PBS. Briefly, 50 µL of sperm extended in TALP at 20 × 10^6^ total sperm/mL were placed on a glass slide and dried at room temperature followed by fixation with 4% paraformaldehyde for 30 min, PBS washing, and permeabilization with Triton X100 0.1% for 15 min at room temperature and then washed again with PBS as described above. Each slide was then incubated with 200 µL of Blocking Buffer (BB) for 2 h at room temperature and then washed with PBS. Primary antibody incubation for detection of capacitation status include the addition of 50 µL of anti-phosphotyrosine antibody (Sigma Cat No: 05–321) at a dilution of 1:50 in BB for ~15 h at 4°C. Slides were then washed with PBS, and 100 µL of secondary antibody (TheromFisher Cat No: A-21052) was added at a dilution of 1:100 in BB for 1 h in the dark followed by a PBS wash. Finally, 50 µL of FITC-PSA (10 µg/mL) diluted in BB 1:10 was added to each slide for the detection of acrosome integrity followed by PBS washing and the addition of 10 µL of DAPI for nuclear staining. Slides were either analyzed immediately or stored at 4°C for future analysis. Four replicates for each bull (2 bulls) were performed with a minimum of 150 sperm per slide enumerated per replicate for a total of 1200 sperm for each treatment. Fluorescence emission and detection for DAPI and FITC were the same as described earlier. For detection of capacitation status, an Alexa Fluor 633 filter was used with excitation and emission 631/650 nm, respectively. Sperm were classified as Intact (green fluorescence due to FITC-PSA binding with the acrosome), Degraded (nuclear DAPI fluorescence and lack of green FITC fluorescence), and Partial degradation (DAPI and FITC detected in merged field) [33,42–46]. Microscopy conditions were the same as described above.

### Genomic DNA (gDNA) isolation from TBT-exposed sperm

DNA was isolated from sperm treated with TBT and VC using a modified protocol from Qiagen Blood and Tissue Kit (60954). All centrifugation was conducted at 17000xg unless otherwise stated. Briefly, 3.5 mL Buffer 1 (150 mM NaCl, and 10 mM ethylenediamine tetraacetic acid) was added to sperm suspensions followed by vortexing for 10 sec at full speed and then centrifugation at 2500xg for 5 min. The supernatant was reduced to ~1 mL and the sample was vortexed again for 10 sec at full speed. Samples were then transferred to new 2.0 mL microfuge tubes with the addition of Buffer 1 (0.5 mL) and back to the empty tube for an additional 10 sec vortex to collect residual sperm. The entire sample was combined and centrifuged for 2 min. The supernatant was discarded and the pellet resuspended in 300 µL of Buffer 2 with the addition of Dithiothreitol (DTT, 1 mM) and 100 uL of Proteinase K (1X) for sample incubation at 55°C for 2 h with intermittent inversion. After incubation, 400 µL of Buffer ATL was added and incubated for 10 min at 55°C. Samples were then passed through DNA binding columns by centrifugation for 1 min. Buffer ATL + ETOH (400 µL each) was added followed by centrifugation for 1 min and the flow through volume was discarded. Samples were placed in a new collection tube and 500 µL of AW1 buffer was added followed by centrifugation for 1 min, and then 500 µL of AW2 buffer was added followed by centrifugation for 3 min. Final elution was accomplished with 50 µL RNase free water. Samples were stored at −20°C for subsequent analyses.

### Enzymatic methyl-seq conversion of genomic DNA isolated from sperm

After DNA isolation, Enzymatic Methyl-seq (EM-seq) Conversion kit module (New England Biolabs #E7125S) was used to convert gDNA cytosines to uridine followed by PCR amplification and downstream epigenomic analyses. Prior to conversion, a DNA integrity number (DIN) was determined (Screen Tape®) for quality control to ensure that all samples met a minimum DIN value of ≥7.5 for downstream sequencing. EM-seq conversion was achieved in a two-step process. The first step included a cocktail consisting of TET2 Reaction Buffer, Oxidation Supplement, DTT, Oxidation Enhancer, TET2, and Fe(II) Solution (containing 2-oxoglutarate) added to 28 µL volumes containing gDNA followed by incubation at 37°C for 1 h. The TET2-converted gDNA samples were purified using AM-Pure beads and final elution performed in 17 µL of supplied buffer. In the second step, DNA was denatured with formamide followed by deamination with APOBEC Reaction Buffer, bovine serum albumin, and APOBEC. Converted gDNA was purified again using AM-Pure beads, and final elution was achieved with 21 uL of Elution Buffer. Converted gDNA was then quantified using a Qubit DNA kit (ThermoFisher Cat No: Q32850). The EM-seq-converted gDNA from sperm were PCR amplified using 20 ng as an input for each PCR reaction and submitted for locus-specific epigenomic analysis using PacBio single-molecule real-time (SMRT) long-read high-fidelity (HiFi) sequencing as described below.

### Whole-genome EM-seq analyses of TBT-treated sperm

Enzymatic detection of 5-methylcytosines (5mC) and 5-hydroxymethylcytosines (5hmC) was achieved with construction of libraries and sequencing by the Genomics Technology Core at the University of Missouri. Libraries were constructed following the manufacturer’s protocol with reagents supplied in the NEBNext® Enzymatic Methyl-seq Kit (New England Biolabs, Catalog #E7120). Briefly, fragmentation of gDNA isolated from TBT-exposed and -unexposed sperm was performed on an M220 sonicator (Covaris). DNA fragments were end repaired/dA-tailed followed by adaptor ligation. A two-step enzymatic conversion protecting 5mC and 5hmC from deamination was performed prior to deamination of the cytosines residues. Fragments were PCR amplified to enrich libraries and incorporate barcodes. The final amplified gDNA constructs were purified by addition of Axyprep Mag PCR Clean-up beads (Axygen), selecting for an insert size of 550 bp. Libraries were quantified with the Qubit HS DNA kit (Invitrogen) and the fragment size analyzed on a 5200 Fragment Analyzer (Agilent). Libraries were diluted according to the standard protocol for sequencing on the NovaSeq 6000 (Illumina). The nf-core/methylseq (v2.6.0) automated bioinformatics pipeline [47] was utilized to analyze enzymatic methylation sequencing (EM-seq) data. This pipeline processes raw FastQ data, aligns reads, and conducts extensive quality control. The Bismark workflow within nf-core/methylseq was used to align reads to the *Bos taurus* genome (assembly ARS-UCD1.3) with Bismark [48]. The methylation coverage files (*.cov.gz) generated by Bismark, which include methylation percentages and read depth at each CpG site, were subsequently analyzed using the methylKit R package for downstream processing [49]. Differentially methylated regions between control and TBT exposed sperm considered significant with an unadjusted *P*-value of ≤0.05 and a ≥20% difference in methylation. Each DMR contained at least three CpG sites in a promoter region [50,51].

### PCR amplification for loci of interest

Three known hypermethylated loci (*PTK2B*, HDAC11, *PAK1*) [52] and three imprinted loci, *(SNRPN, H19, KCNQ1)* were selected from the bovine sperm genome to quantify changes to methylation status in response to TBT exposure (Table 1). Primers were designed for EM-seq-converted DNA using the Meth Primer program to amplify the promoter regions of each gene (genome.ucsc.edu). PCR amplification within 20 µL reactions consisted of 20 ng of methyl-converted gDNA from sperm, 10 µL of Go Taq Hot-Start Green Master mix (Promega, Cat No. M5122), 0.2 µM each of forward and reverse primers, and water. PCR conditions were as follows: 95°C for 3 min, followed by 35 cycles of 95°C for 30 s, 59°C for 30 s, 72°C for 30 s, and final extension at 72°C for 7 min. Successful amplification was confirmed by gel electrophoresis on a 1% agarose gel containing 0.1% ethidium bromide electrophoresed at 90 V for 80 min followed by a 2 ms exposure on a UV transilluminator. The remaining volume of each PCR product was purified using the QIA quick PCR purification kit (Qiagen, Cat. No: 28104) according to the manufacture specified protocol.

**Table 1.** Hypermethylated regions in bovine sperm selected for PCR amplification following exposure to TBT.

Gene	Accession #	Gene ID	Methylation (%)	FORWARD PRIMER (5’– 3’)	REVERSE PRIMER (3’– 5’)
PAK1	NM_001076898	533729	48.1 ± 13.1	TGGTGTAATTTTAAGAGTTATTAATTGTTG	AAAACCACAAAAAAACCCCAC
HDAC11	NM_001102056	519899	89.3 ± 3.0	TGTGATGTTTGAATTTTTAATTTTG	CAACCCAACTATACCAACATATACC
PTK2B	NM_001102252	541008	66.6 ± 8.7	TAGGTGGGTGTGGGTAAAGTTATTA	ACCACCACCATAAACTCTAAAAAAC
**IMPRINTED**					
H19	NR_003958	100126192	80 ± 10	GATAGGGTGGGGATTTAGGATAG	TAACCAAAAACATCAAAAAAACAAC
SNRPN	NM_001079797	780877	70 ± 10	TAGGTGGGTGTGGGTAAAGTTATTA	ACCACCACCATAAACTCTAAAAAAC
KCNQ1	NM_001205441	784876	70 ± 10	TAAGGTGGAGTTGTTTATGAAATTG	CCTAAACCCCCTAAAACCTAACTAC

Gene ID, accession numbers, percent methylation, and list of primers used for amplification.

### Locus-specific methylation analyses of TBT-treated sperm

Purified PCR amplicons obtained from EM-seq-converted gDNA from sperm treated with TBT 10 nM and VC were submitted for PacBio single-molecule real-time (SMRT) long-read high-fidelity sequencing for increased robustness of detection of methylation status (*n* = 2 bulls, 4 pooled independent reps/bull). The PCR products were sequenced on PacBio Sequel IIe SMRT platform using Interdisciplinary Center for Biotechnology Research (ICBR) facility at the University of Florida. Circular consensus sequencing (CCS) mode was used to generate HiFi reads with filtering for ≥3 passes per individual polymerase read. HiFi read mapping and visualization of DNA methylation states, including non-supervised clustering, at the single-molecule level were conducted using MethylMapper [53]. Reads containing more than 5% non-converted CH (H, any base but G) sites were excluded.

### Functional analyses of DMRs

Common DMRs revealed by EM-seq in both TBT treatments (100 nM and 10 nM) compared to unexposed sperm were analyzed using Ingenuity Pathway Analysis (IPA), Qiagen (IPA 4.0; *n* = 1 bull, 4 pooled replicates/treatment). Criteria for the set of common DMRs used in each analysis was determined based on an unadjusted *P*-value of ≤0.05 with ≥3 CpG sites in sequenced promotor regions using multiple comparisons correction (i.e., −log(Benjamini–Hochberg *P*-value) ≥1.3 or Benjamini–Hochberg *P*-value ≤0.05) that were predicted as increased or decreased based on a *Z*-score ≥2.0 and ≤−2.0, respectively. The predictions were deemed to be activated or inhibited based on the *Z*-score criteria described above. IPA mapped the input genes to knowledge bases and identified the most relevant biological functions, networks, and canonical pathways related to the altered methylation profiles in the treatment and control. Expression analysis was designated as the core analysis type, and Expr Fold Change indicated as the measurement type. The top canonical pathways associated with methylation difference are presented [54–58].

### Gene ontology and molecular function analysis

Gene ontology enrichment analysis was performed using the tool GO PANTHER version 19.0 (https://pantherdb.org/) to better understand the functional roles of genes with common DMRs between both TBT exposure levels (100 nM and 10 nM) compared to unexposed control sperm. Genes were uploaded into the Protein Analysis Through Evolutionary Relationships (PANTHER) classification system. The gene list analysis option was used to identify overrepresentation of gene ontology (GO) terms in the gene list data. The most significantly enriched ontologies were identified based on the list of molecular functions to which these genes were assigned [59–61].

### Statistical analysis

Sperm kinematic, TUNEL, acrosome, and capacitation data were analyzed by logistic regression using a generalized linear mixed effect model with SAS 9.4 software (St. Louis, Missouri, USA). Treatment and time were considered fixed effects while bull and replicate were assigned as random effects. Parameters were evaluated for normality (Shapiro–Wilk test). Square root and logarithmic (base 10) transformations were applied to variables that did not meet the normality assumption (*p* ≤ 0.05). Parameters expressed as percentage were arcsine transformed. Post-hoc analyses were performed by using Tukey’s HSD (*p* ≤ 0.05). All data are presented as the back transformed value. For sequencing analyses, DMRs between control and TBT-exposed sperm were calculated based on % methylation in promoter regions by paired *t*-test of normally distributed data. Criteria for significance of DMRs between treatments included a minimum of three CpG sites within a promoter region and ≥20% difference in methylation with an unadjusted *P*-value of ≤0.05.

## Results

### TBT exposure to postejaculatory sperm increases DNA fragmentation

**Figure 1.**
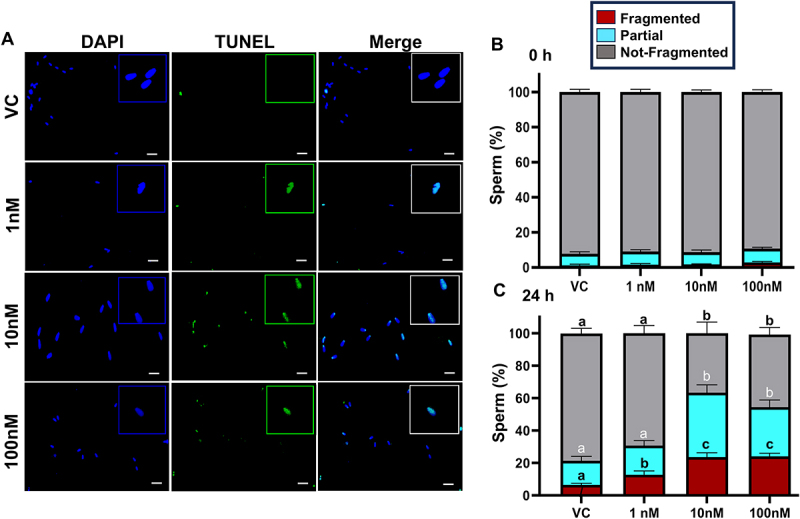
TBT exposure affects the DNA integrity of postejaculatory sperm. Frozen-thawed bovine sperm were subjected to a TUNEL DNA fragmentation assay following exposure to TBT (0, 10, 100 nM) for 24 h (A). Nuclear staining was detected by DAPI (blue), while DNA fragmentation (FITC, green) was categorized as fragmented, partially fragmented, and not fragmented. DNA fragmentation was categorized immediately after initial exposure of TBT (B −0 h), and again after 24 h (C). *n* = 2 bulls; 4 individual replicates/bull. *p* ≤0.05; scale bar = 20 μm. Different superscripts (^a,b,c,d^) indicate *p* ≤0.05 between treatments and within respective time points.

The effects of TBT (1, 10, and 100 nM) exposure for 24 h on DNA integrity of postejaculatory sperm were quantified by DNA fragmentation assay (TUNEL) at 0 and 24 h following the addition of TBT to sperm under non-capacitating conditions. DNA fragmentation was categorized as fragmented, partially fragmented, or not fragmented (Figure 1). TBT had no effect on DNA fragmentation at 0 h (*p* = 0.58), but TBT exposure to sperm for 24 h elicited loss of DNA integrity across all fragmentation categories between treatments (*p* < 0.05). The percentage of sperm with complete DNA fragmentation was higher in sperm exposed to TBT 100 nM (25.2 ± 2.8) and 10 nM (23.6 ± 2.8) compared to 1 nM (12.7 ± 1.5) and VC (6.4 ± 1.64).

### Acrosome integrity is compromised following sperm exposure to TBT

**Figure 2.**
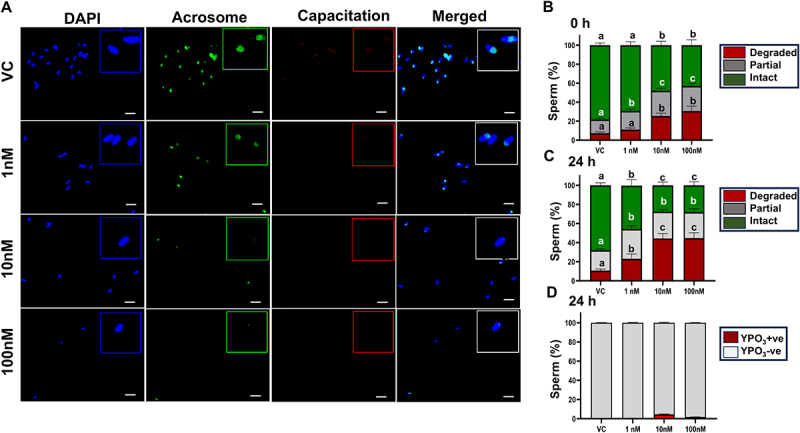
Acrosome integrity is compromised by TBT exposure to postejaculatory sperm. Frozen-thawed bovine sperm were subjected to immunocytochemistry for detection of acrosome integrity (FITC-PSA-green) and capacitation status (YPO3-red), along with nuclear staining (DAPI-blue) in a non-capacitating medium (A). Quantification of acrosome integrity was categorized as degraded, partially degraded, or intact at 0 h and 24 h (B-C) following exposure to TBT (1, 10, 100 nM). Capacitation status was also evaluated after 24 h (D). *n* = 2 bulls; 4 individual replicates/bull. Scale bar = 20 μm. Different superscripts (^a,b,c,d^) indicate *p* ≤0.05 between treatments and within respective time points.

Postejaculatory sperm were subjected to immunofluorescence for detection of acrosome integrity (FITC-PSA) and capacitation status for detection of tyrosine phosphorylation (YPO3) following exposure of TBT for 24 h (Figure 2(A)). Quantification of acrosome integrity was categorized as degraded, partially degraded, or intact at 0 and 24 h. The percentage of sperm with completely degraded acrosomes was significantly higher in 100 nM (30.6 ± 4.6) and 10 nM (25.4 ± 4.04) TBT groups as compared to 1 nM (11.1 ± 3.5) and VC (7.4 ± 2.6) samples at 0 h (*p* = 0.004, Figure 2(B)). Loss of acrosome integrity persisted at 24 h across all treatments of TBT-exposed sperm compared to VC (*p* < 0.05) (Figure 2(C)). The capacitation status of TBT-exposed postejaculatory sperm was also evaluated at 0 and 24 h. Unlike acrosome integrity, exposure of sperm to TBT did not alter the tyrosine phosphorylation status of sperm (Figure 2(D)).

### Sperm kinematics are unaltered following 24 h of exposure to TBT

**Figure 3.**
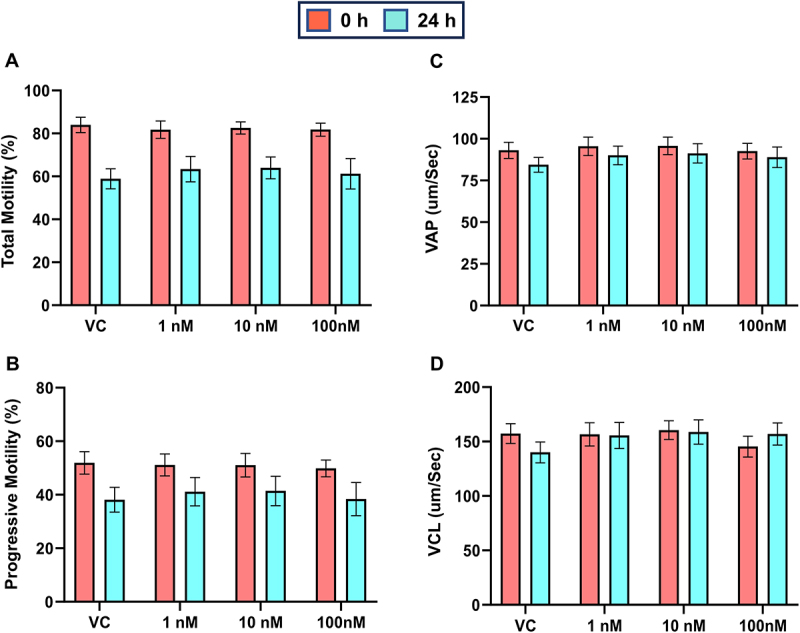
TBT exposure to postejaculatory sperm for 24 h does not affect sperm kinematics. Bull sperm was exposed to TBT (0-100 nM) for up to 24 h at ambient temperature in non-capacitating conditions. Sperm kinematics were quantified by computer-assisted sperm analyses to determine (A) total and (B) progressive motility. The average-path velocity (VAP), and curvilinear velocity (VCL) of sperm samples was also determined, respectively (C-D). *n* = 2 bulls, 4 individual replicates/bull.

The effects of TBT (1, 10, and 100 nM) on sperm motility kinematics were quantified by CASA at both 0 and 24 h of exposure (Figure 3). Sperm kinematics parameters included total motility (TM), progressive motility (PM), average path velocity (VAP), and curvilinear Velocity (VCL). Treatment had no significant effect on any sperm kinematics parameters observed at either 0 or 24 h of exposure.

### Exposure of sperm to TBT affects DNA recovery yields pre- and post-enzymatic-methyl conversion

DNA was isolated from sperm following 24 h of exposure to TBT (1, 10, and 100 nM) for enzymatic-methyl conversion (EM-seq) and PCR amplification of selected loci. DNA was eluted in equal volumes and yield depicted as concentration of DNA (ng/µL) was significantly lower in all TBT treated sperm compared to VC (Supp Figure S1, *p* ≤ 0.05). TBT 1 nM (34.1 ± 4.4) and 10 nM (35.0 ± 1.3) had higher DNA concentration than 100 nM (26.1 ± 2.9) treated groups (*p* < 0.05) (Supp Figure S1A). Consequently, the yield (ng/µL) of enzymatic methyl-converted DNA was lower in TBT exposed groups compared to unexposed (VC) sperm (*p* < 0.05) (Supp Figure S1B). PCR amplification of methyl-converted DNA from six loci (*PTK2B, SNRPN, PAK1, HDAC11, KCNQ1, H19*) was performed for sperm treated with TBT 10 nM and VC (Supp Figure S1C). These loci were selected for presumed susceptibility to epigenetic modifications due to predominantly hypermethylated regions [52] (Table 1).

### TBT exposure to sperm affects the methylation of genome-wide promoter regions

**Figure 4.**
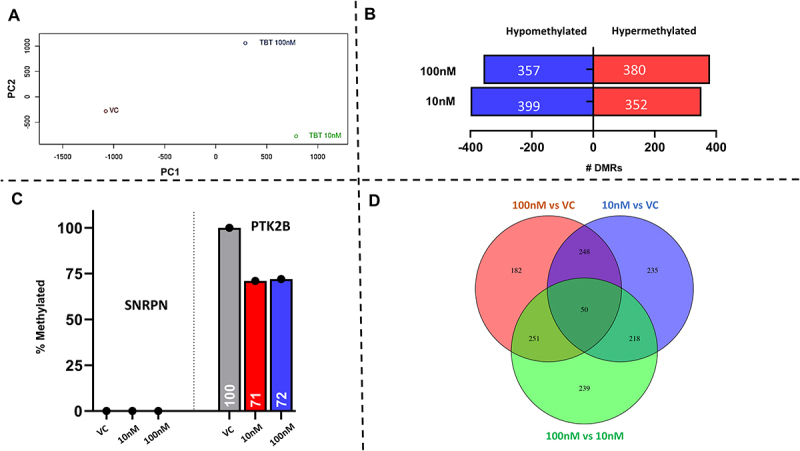
TBT exposure alters the genome-wide methylation status of mature sperm. Whole-genome Em-seq was performed to identify changes to the methylation landscape of postejaculatory bull sperm exposed to 10 nM and 100 nM TBT for 24 h compared to vehicle control (VC − 0.1% DMSO). Principal component analysis of TBT 10 nM, 100 nM, and VC-exposed sperm in which all three groups form distinct clusters (A). Total numbers of DMRs between 100 nM TBT vs. VC and 10 nM TBT vs. VC (B). TBT exposure resulted in 29% demethylation within the promotor region of *PTK2B*, whereas that of *SNRPN* (imprinted) remained unaffected (C). Venn diagram of differentially methylated regions between 100 nm TBT and VC, TBT 10 nM and VC, and 100 nM TBT and 10 nM TBT (D). *n* = 1 bull; 4 pooled replicates/treatment.

**Figure 5.**
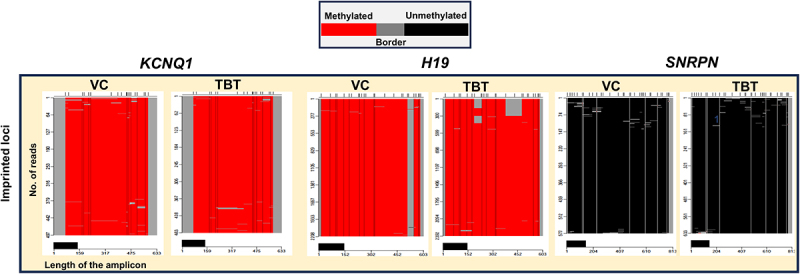
Select loci from mature sperm exposed to TBT for 24 h do not show changes in DNA methylation. Sperm from two different bulls were exposed to 10 nM TBT or VC (0.1% DMSO) for 24 h. Promoter regions of the indicated genes were PCR amplified from converted/deaminated gDNA and subjected to SMRT sequencing. Heatmaps depict CpG methylation of the indicated number of sequenced single molecules (x-axis). Red indicates two or more consecutively methylated cytosines, black indicates two or more consecutively unmethylated cytosines, and gray indicates transitions between methylated and unmethylated cytosines. The promoter regions of imprinted loci *(KCNQ1*, *H19*, *and SNRPN*) were unaffected by TBT treatment. *n* = 2 bulls; 4 pooled replicates/treatment.

Principal component analyses (PCA) of whole-genome EM-seq of promoter sequences of sperm indicate that TBT-exposed samples (10 nM and 100 nM) and unexposed vehicle control sperm (VC) form three distinct clusters, suggesting the presence of differentially methylated regions (DMRs) (Figure 4(A)). Comparison of sperm exposed to TBT *versus* VC revealed ~750 DMRs, irrespective of the exposure level (Figure 4(B)). Compared to VC, 100 nM TBT induced 357 hypomethylated regions and 380 hypermethylated regions, whereas 10 nM TBT resulted in 399 hypomethylated regions and 352 hypermethylated regions. In agreement with promoter-specific EM-seq followed by SMRT amplicon sequencing (Figure 5), TBT exposure did not affect the methylation level of imprinted *SNRPN* but caused a reduction in methylation of the *PTK2B* promoter as compared to control (Figure 4(C)). The genome-wide results identified DMRs between VC sperm and sperm exposed to 10 nM TBT and 100 nM TBT. A total of 182 DMRs are unique to the comparisons of 100 nM TBT *vs*. VC, 235 (10 nM TBT *vs*. VC), and 239 (100 nM TBT *vs*. 10 nM TBT). In addition, 268 DMRs were shared between the 10 nM TBT and 100 nM TBT conditions, and 50 DMRs overlap in all three treatment comparisons (Figure 4(D)).

### DNA methylation of select, imprinted promoter regions remains unaffected by TBT

**Figure 6.**
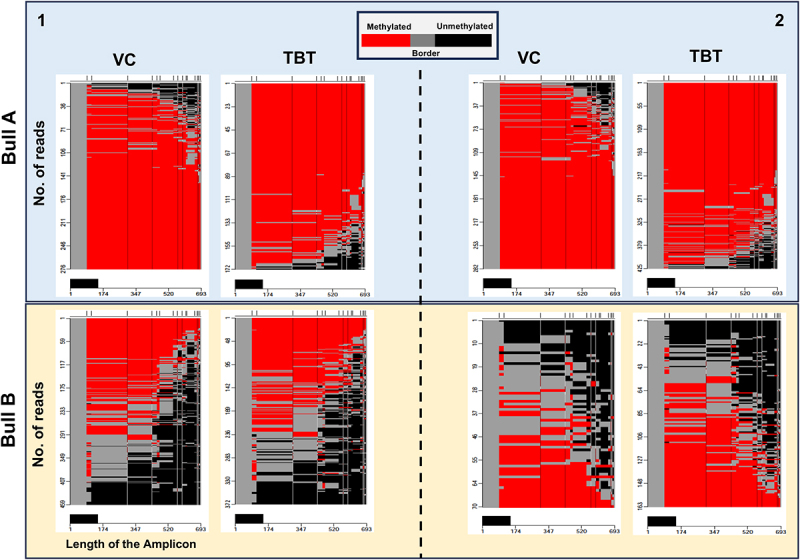
Methylation analyses of the *PAK1* promotor region of sperm from two different bulls exposed to TBT. Postejaculatory sperm from two different bulls were exposed to 10 nM TBT and VC for 24 h in duplicates. The promoter region of the *PAK1* gene was PCR amplified from converted/deaminated gDNA and subjected to SMRT sequencing. Heatmaps depict CpG methylation of the indicated number of sequenced single molecules (x-axis). Red indicates two or more consecutively methylated cytosines, black indicates two or more consecutively unmethylated cytosines, and gray indicates transitions between methylated and unmethylated cytosines. The left panel (1) indicates independent replication from the right panel (2). Note the similar pattern but different levels of CpG methylation of *PAK1* from each bull regardless of TBT exposure. *n* = 2 different bulls and two independent replicates each per treatment (TBT and VC).

The locus-specific DNA methylation of CpG sites in promoter regions was determined for amplified, select loci using PacBio single-molecule real-time (SMRT) long-read high-fidelity sequencing. Locus-specific methylation changes to the promoter regions of select imprinted (*KCNQ1, H19, SNRPN*) and hypermethylated (*PAK1)* regions were quantified to identify differential amounts of CpG methylation among two individual males. Heatmaps depict CpG methylation level of single molecules, which correspond to individual sperm. The loci of imprinted genes (*KCNQ1*, *H19*, *SNRPN*) remained largely unaffected by TBT exposure (Figure 5). However, the *PAK1* promoter region shows a distinct methylation pattern with each bull exhibiting markedly different CpG methylation levels (Figure 6).

## Functional analyses of DMRs associated with TBT sperm exposure include embryo development, cell signaling, and immune response

**Figure 7.**
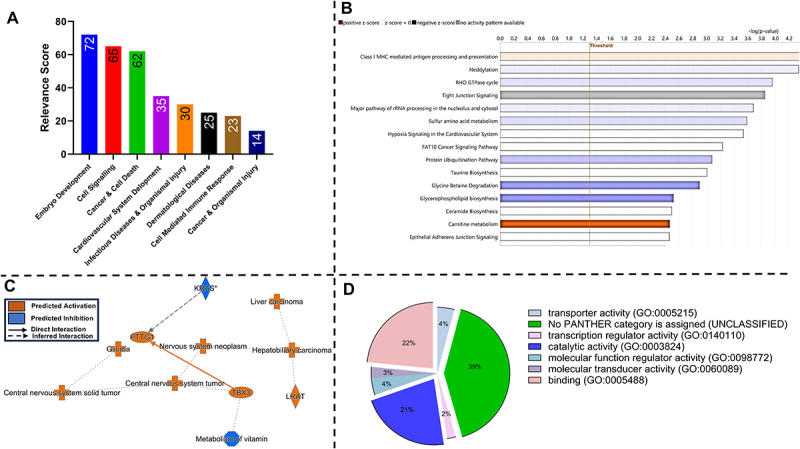
Molecular function and gene ontology of TBT-induced DMRs that were commonly differentiated between 100 nM and 10 nM sperm exposure for 24 h. Characterization of bio-function categories by relevance and significance score where canonical pathways and function annotations were classified (A). Activation or inhibition *Z*-score of the top 10 most significant canonical pathways (B). Orange indicates activated pathway, blue is inhibited, white means no activity, and gray indicates that no activity pattern could be measured. Graphical summary of IPA for TBT and VC groups indicating inhibition of cell signaling by the KRAS pathway, nervous system, and tumor events from TBT exposure (C). Orange symbol and arrows = activation; blue symbol and arrows = inhibition; solid line = direct effect and dashed line = indirect effect. Molecular functions of common TBT-affected DMRs and VC as identified by panther include binding (22%), catalytic activity (21%), and unclassified categories (39%) (D). *n* = 1 bull; 4 pooled replicates/treatment.

IPA analyses identified eight bio-function and disease categories based on relevance score as a result of TBT exposure altering DMRs within the promoter regions of mature sperm. The major categories which were identified included embryo development, cell signaling, cancer, and cell death (Figure 7(A)). Canonical pathway analysis with common DMRs between both treatments and control that were mapped by IPA led to 65 significant pathways. The top 15 most significant pathways presented were mostly related to immune response, cell signaling, and cell cycle and cell death (Figure 7(B)). Network analyses of affected DMRs indicated that KRAS is the major pathway predicted to be inhibited by TBT leading to compensatory activation of PTTG1 and TBX3 transcription through inhibition of vitamin metabolism, thereby promoting nervous system neoplasms, carcinomas, and tumor activity (Figure 7(C)). Gene ontology and molecular function enrichment analysis using GO Panther indicated that common identifiable DMRs were mainly related to the functional categories of binding (22%) and catalytic activity (21%), while the largest proportion (39%) were not assigned due to lack of annotation (Figure 7(D)).

## Discussion

Impacts of environmental stressors on male factor infertility are largely focused at the testicular level where disruptions to cellular processes during spermatogenesis are presumably most susceptible due to increased exposure time, incomplete chromatin compaction, and active transcription or translation. In contrast, postejaculatory mammalian sperm have unique cellular and structural characteristics that protect sperm during transit within the female reproductive tract by limiting access to open chromatin and removing impacts on organelles that are absent in the quiescent spermatid. Despite these protective attributes of mature sperm, susceptibility to the structural, genomic, and epigenomic integrity from environmental stressors present in seminal plasma and the female reproductive tract remain largely unexplored. Experiments herein utilized a bovine model to determine putative impacts of the organotin TBT on postejaculatory sperm function. The bovine is a highly informative model to study male infertility and human embryo development as it overcomes gamete limitations of ethical constraints associated with human research [62,63]. Importantly, postejaculatory sperm increases physiological relevance compared to testicular sperm while lending reproducibility by reducing or eliminating inter and inter-ejaculate variability. Human and bovine mature sperm also contain about 8% euchromatin, lending compatibility for genomic and epigenomic modeling [17,15]. Similarities between bovine and human germline development revealed by single-cell RNA sequencing also support the bovine as a translational model for human [64]. There are additional likenesses between human and bovine sperm morphology [65–68], insemination location [67], use in assisted reproductive technologies such as in vitro fertilization [32], and the sperm transcriptome [13,68].

Despite studies to quantify TBT in reproductive fluids, concern for human exposure is attributed to environmental persistence and stability, where detection in serum (0.59–11.8 ng/mL), whole blood in Michigan (64–155 ng/mL) and urine from exposed workers has been reported [69,70]. A 24 h exposure time to TBT was used to mimic temporal aspects of sperm transit after semen deposition in the female reproductive tract. Exposure of sperm to a range of environmentally relevant concentrations of TBT resulted in no detectable change to sperm motility and kinematic parameters at both 0 and 24 h. These findings alone are significant because sperm motility and kinematics are the most commonly used traits to assess sperm quality and most reliable indicators of fertility. Initial screening of samples suggested no observable impact on sperm motility or capacitation status, but further analyses detected an immediate effect on acrosome integrity. Exposure to TBT resulted in acrosome degradation within 10 min of co-incubation (0 h), suggesting that even short-term exposure to OTs such as TBT in seminal fluid or the female reproductive tract may be sufficient to elicit changes likely to impact sperm penetration through the zona pellucida. Indeed, previous experiments conducted by our group determined that short-term exposure of sperm to TBT (4 h) was sufficient to significantly reduce embryo cleavage and prevent blastocyst development using an in vitro fertilization model similar to human clinical settings [11]. Loss of acrosome integrity is physiologically preceded by capacitation that is initiated within the oviduct. The impact of TBT on acrosome integrity but not capacitation appears autonomous and thus likely due to direct impacts on structural function rather than classical activation of pathways associated with capacitation due to no observable changes in tyrosine phosphorylation when sperm were maintained in non-capacitating conditions. Collectively, these findings may prove beneficial for identifying appropriate assisted reproductive procedures for human clinical applications such as intracytoplasmic sperm injection (ICSI) that can be applied to overcome structural defects such as loss of acrosome integrity that prevents sperm penetration during gamete interaction.

An additional attribute of TBT is the demonstrated ability to interact and bind DNA [22,71–74]. More specifically, TBT has been shown to bind DNA of salmon sperm with strong affinity [75,76], albeit with unknown consequences on downstream effects following fertilization and subsequent embryo development. The bovine model herein clearly demonstrated a negative effect on sperm exposure to TBT with significant reductions in DNA integrity of sperm after 24 h. Notably, these changes were not observed at initial (0 h) analyses. Furthermore, isolation of DNA from TBT exposed sperm was also negatively impacted with samples of higher TBT exposure resulting in less total DNA recovery and predictably, less recovery following EM-seq conversion. Intuitively, the data suggest that an explanation for decreased DNA recovery may be due to toxicity and therefore loss of viability. However, the total motility of sperm was not impacted after 24 h exposure to TBT in any of the treatment groups, and sperm motility is highly associated with viability [77–80]. Therefore, the reduction in quantifiable DNA following increased exposure to TBT is particularly concerning from a clinical perspective because changes to the genomic integrity of sperm appear uncorrelated to motility and may be overlooked as an explanation for idiopathic subfertility.

Recent traction on the importance of the sperm epigenome has provided valuable insight into the functions of miRNAs and other non-coding RNA – associated mechanisms important for post-fertilization events [81–85]. The sperm epigenome plays a crucial role in many biological processes including early embryo development, zygotic genome activation, imprinted clusters, and X chromosome inactivation [17,25,26,86,87]. Environmental factors that alter DNA methylation, non-coding RNAs, and histone modifications may impact embryo development with transgenerational effects [88–93].

TBT is a well-described epigenetic modifier known to induce changes to the methylome in somatic cells [22,94–97]. Environmental factors that induce epigenetic modifications to mammalian sperm have been described during spermatogenesis [9,10,98–105], but studies to determine potential effects on postejaculatory sperm have not been described. The present study utilized a comprehensive genome-wide methylation analysis of postejaculatory bovine sperm to determine effects of TBT on the sperm methylome. Sperm exposed to 10 nM and 100 nM TBT for 24 h were separated from each other and from control samples in PCA space. These changes included ~ 750 DMRs as a result of direct exposure to TBT. Such findings are striking as they are the first evidence of alterations to the methylation landscape of postejaculatory sperm.

Methylation analysis of *PTK2B* at the whole genome level indicated a reduction in the promoter region methylation. Additionally, 268 DMRs were shared between the 10 nM TBT and 100 nM TBT treatments, which may be attributed to the direct effect of gene-specific methylation changes on biological networks rather than individual genes. IPA analysis indicated that identified DMRs affected by TBT were mainly associated with mammalian embryo development, cell signaling, cancer, and cell death. More specifically, inhibition of TBX3 activity presents meaningful implications, as TBX3 drives cell fate decisions in early embryos. Additionally, dsRNA knockdown of TBX3 in porcine embryos reduces 4-cell to blastocyst formation rates, ZGA expression, acetylation of histone H3K9 and H3K27, SCAN4 protein, and DNMT1, as well as increases in 5-methycystosine (5mC) and DNA damage [106]. TBX3 knockdown also increased *NANOG* and *OCT4* expression in blastocysts, which is required for normal blastocyst development in bovine and humans [107–109].

In complement to genome-wide methylation sequencing, six loci in postejaculatory bovine sperm (*PTK2B*, *HDAC11*, *PAK1*, *SNRPN*, *KCNQ1*, *and H19*) were identified as predominately hypermethylated and utilized to determine if TBT exposure altered the methylation profile in promoter regions. Results from SMRT long-read PacBio sequencing indicated that imprinted loci (*H19, SNRPN, KCNQ1*) and *PAK1* were unaffected by TBT treatment in amplified regions. However, methylation analysis of the *PAK1* promoter region illuminates the effect of individual males on the methylation profile at that locus, demonstrating that the methylation landscape forms a distinct pattern of CpG methylation for each bull. Quantitative analyses of *PTK2B* and *HDAC11* did not appear as conclusive as *PAK1*, some of which may be attributed to sequencing difficulty.

Results from experiments herein introduce a paradigm-shift to understanding environmental perturbations to the sperm epigenome. While the potential influence on downstream embryo development remains unknown, modifications to single CpG islands in ram sperm revealed impacts on growth, development, male infertility, neurodevelopment, and fetal reprogramming [110,111]. Direct mechanisms of TBT as an epigenetic modifier in neural cells are attributed to decreased transcript abundance of TET gene transcripts [22]. Although not explicitly determined within these experiments, such a classical explanation is not likely due to lack of transcription and translation in the mature sperm. However, mammalian sperm do indeed express TETs and DNMTs [112–115], providing insight into the interaction with TBT as a potential mechanism of alterations to the mature sperm methylome. Further studies to determine putative carryover effects of epigenetic modifiers to mature sperm are likely to include implications on embryo development and consequences of environmental impacts on the sperm epigenome.

## Conclusions

Postejaculatory sperm exposure to tributyltin chloride for 24 h does not alter commonly assessed traits associated with male factor infertility such as sperm motility and kinematics. However, TBT negatively impacts acrosome and DNA integrity without altering the capacitation status of sperm. TBT also elicits methylomic changes in mature sperm, suggesting a paradigm-shift to our understanding of how environmental stressors may alter the epigenetic landscape of sperm during transit within the female reproductive tract. Collectively, these data present initial evidence for alterations to the postejaculatory sperm epigenome from exposure to a known EDC and epigenetic modifier.

## Abbreviations

TBT: tributyltin chloride
EDC: endocrine disrupting chemical
DMRs: differentially methylated regions
CASA: computer-assisted sperm analysis
OT: organotin
DAPI: 4,’6-diamidino-2-phenylindole
IPA: Ingenuity Pathway Analysis
DNMTs: DNA methyltransferases
TETs: ten-eleven translocation
TM: total motility
PM: progressive motility
VCL: curvilinear velocity
VAP: average path velocity
ATL: tissue lysis buffer
AW: wash buffer
KRAS: Kirsten rat sarcoma virus
PTTG1: pituitary tumor-transforming 1 protein (*securin*)
TBX3: T-Box Transcription Factor 3

## Supplementary Material

Supplemental Material

## Data Availability

All data will be made available upon reasonable request. The data discussed in this publication have been deposited in NCBI’s (Gene Expression Omnibus) and are accessible through GEO Series accession number GSE298065 (https://www.ncbi.nlm.nih.gov/geo/query/acc.cgi?acc=GSE298065).
