# Supplementary material for: Tributyltin chloride alters the structural, genomic, and epigenomic integrity of postejaculatory mammalian sperm
Source: Epigenetics. 2025 Sep 15;20(1):2552129. doi: 10.1080/15592294.2025.2552129 (PMC12439583; doi:10.1080/15592294.2025.2552129)
Supplement: Supplemental Material [file KEPI_A_2552129_SM8265.zip › Supplementaty files/Supplemental Figure caption.docx]

**Supplemental Figure 1. DNA and enzymatic methyl-conversion yield of TBT exposed sperm subjected to PCR amplification of methyl-converted DNA**. Exposure of postejaculatory frozen-thawed bovine sperm to TBT for 24 h resulted in recovery of lower quantities of DNA **(A)**, and subsequently lower recovery after enzymatic-methyl conversion **(B)**. PCR amplification of six respective loci (*PTK2B, SNRPN, PAK1, HDAC11, KCNQ1, H19*) for vehicle control (VC) and 10nM TBT treatment in duplicate.
