# Supplementary figures and images for: Tributyltin chloride alters the structural, genomic, and epigenomic integrity of postejaculatory mammalian sperm
Source: Epigenetics. 2025 Sep 15;20(1):2552129. doi: 10.1080/15592294.2025.2552129 (PMC12439583; doi:10.1080/15592294.2025.2552129)

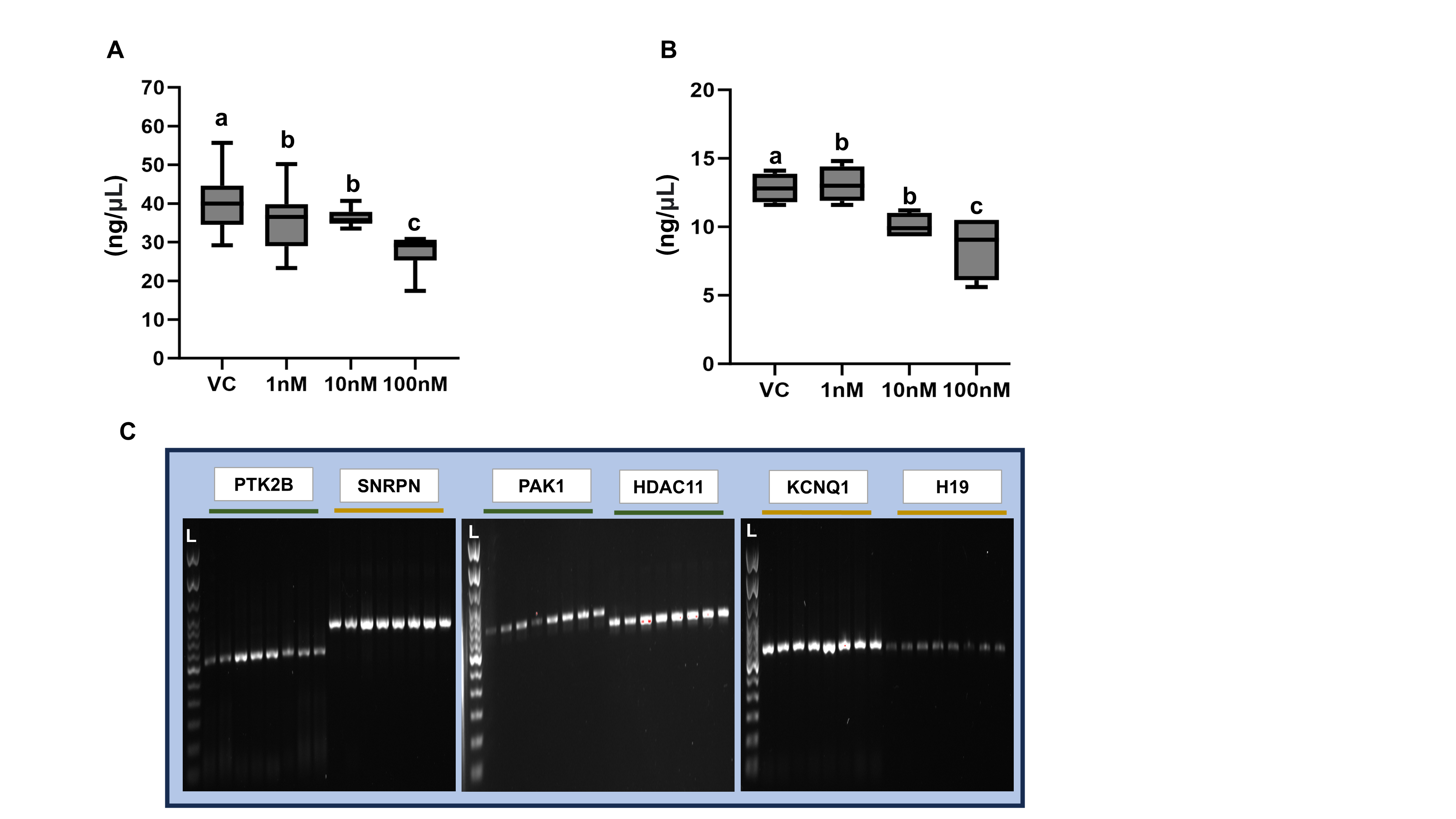

Supplement: Supplemental Material [file KEPI_A_2552129_SM8265.zip › Supplementaty files/Suppl Figure 1.tif]
